# Artificial Intelligence in *Clinical and Translational Science*: From Bench Insights to Bedside Impact

**DOI:** 10.1111/cts.70383

**Published:** 2025-11-03

**Authors:** Mohamed H. Shahin, Qi Liu

**Affiliations:** ^1^ Pfizer Research & Development Groton Connecticut USA; ^2^ Office of Clinical Pharmacology, Office of Translational Sciences, Center for Drug Evaluation and Research U.S. Food and Drug Administration Silver Spring Maryland USA

**Keywords:** artificial intelligence, drug development, large language models, machine learning

The healthcare and life sciences sectors are experiencing a transformative moment that cannot be overlooked. Our biological understanding, technology, and data are coalescing to leverage unprecedented opportunities for innovation [1, 2]. At the center of this transformation lies Artificial Intelligence (AI) and Machine Learning (ML), which have advanced from speculation to working technologies that can make actual differences in patient care and drug development [3, 4]. Only a few years ago, AI was framed in the context of its potential in clinical pharmacology, drug discovery, and development. Following the 2024 Nobel Prize in Chemistry, which was awarded for the AI‐based prediction of protein structure, it became increasingly difficult to ignore the scientific merit of the technology [5]. We are now experiencing implementations that are changing how we approach these disciplines [6, 7, 8]. We have transcended previous discussions about whether AI will help and are asking more nuanced questions about how we deploy these technologies in a responsible manner, such that they deliver reliable and reproducible results, and produce meaningful value in clinical and translational research.

The AI‐themed issue in Clinical and Translational Science addresses exactly these questions by collating different viewpoints from around the field and providing substantive evidence to demonstrate the breadth of the current AI applications that are transforming the practice of clinical and translational sciences (Table 1). From the earliest phases of drug discovery through design and optimization of clinical trials, from developing personalized treatment approaches to monitoring drug safety postmarket approval, and to collecting real‐world evidence—these contributions illustrate the current state of the art in utilizing AI in drug discovery and development and also characterize our current capabilities, while providing a vision for future innovation within clinical and translational sciences.

**TABLE 1:** Brief summary of manuscripts in the *clinical and translational science* AI‐themed issue.

#	Title	Manuscript type	Objective	Key highlights	Potential AI application	References
1	Predicting Pharmacokinetics in Rats Using Machine Learning: A Comparative Study Between Empirical, Compartmental, and PBPK‐Based Approaches	Original Research Article	Compare ML with traditional PK modeling for rats	ML achieved comparable accuracy to PBPK for PK prediction	Preclinical PK prediction without full mechanistic data	[9]
2	Large Language Models and Their Applications in Drug Discovery and Development: A Primer	Review	Introduce LLM concepts and applications in drug development	Clear taxonomy of LLM use cases from discovery to postmarketing	Literature mining, protocol drafting, hypothesis generation	[4]
3	AI In Action: Redefining Drug Discovery and Development	Perspective	Overview of AI applications in drug R&D	Use cases in molecule generation, lead optimization, and trial simulation	AI‐accelerated drug discovery platforms	[10]
4	Variational Autoencoders for Generative Modeling of Drug Dosing Determinants in Renal, Hepatic, Metabolic, and Cardiac Disease States	Tutorial	Use VAEs for dosing modeling in disease states	Generated realistic dosing patterns	Simulated dose–response exploration	[11]
5	Machine Learning Framework to Predict Pharmacokinetic Profile of Small Molecule Drugs Based on Chemical Structure	Original Research Article	Predict PK profile from molecular structure	High throughput with minimal wet lab data	Early PK screening in discovery	[12]
6	Nontargeted Metabolomics for the Identification of Plasma Metabolites Associated with Organic Anion Transporting Polypeptide 1B1 Function	Original Research Article	Identify plasma metabolites linked to OATP1B1	Combined metabolomics and AI analysis	Biomarker identification for transporter function	[13]
7	Machine Learning Identifies Fatigue as a Key Symptom of Fibromyalgia Reflected in Tyrosine, Purine, Pyrimidine, and Glutaminergic Metabolism	Original Research Article	Identify metabolic pathways linked to fatigue	Tyrosine, purine, pyrimidine pathways implicated	AI‐guided biomarker discovery	[14]
8	Leveraging In Silico and Artificial Intelligence Models to Advance Drug Disposition and Response Predictions Across the Lifespan	Review	Summarize in silico and AI models for predicting drug disposition and response across age groups	Integration of PBPK, QSP, and AI to predict PK/PD in special populations	Age‐specific drug dosing optimization and simulation	[15]
9	AI‐Driven Applications in Clinical Pharmacology and Translational Science: Insights From the ASCPT 2024 AI Preconference	Review	Summarize key AI trends discussed at ASCPT 2024 AI preconference	Highlights AI in PK/PD modeling, trial design, and regulatory science	Cross‐domain integration of AI in clinical pharmacology	[8]
10	MoLPre: A Machine Learning Model to Predict Metastasis of cT1 Solid Lung Cancer	Original Research Article	Develop ML model for lung cancer metastasis prediction	ML model showed high accuracy using imaging and clinical features	Early cancer progression prediction for trial enrichment	[16]
11	A Tutorial and Use Case Example of the eXtreme Gradient Boosting (XGBoost) Artificial Intelligence Algorithm for Drug Development Applications	Tutorial	Demonstrate XGBoost applications in drug development datasets	Walkthrough of hyperparameter tuning and model interpretation	Biomarker‐based patient stratification	[17]
12	Exploration of Using an Open‐Source Large Language Model for Analyzing Trial Information: A Case Study of Clinical Trials With Decentralized Elements	Original Research Article	Apply open‐source LLM to analyze decentralized trial data	Identified operational insights and trial element classification	Automated trial design review and DCT readiness checks	[18]
13	Agents for Change: Artificial Intelligent Workflows for Quantitative Clinical Pharmacology and Translational Sciences	Review	Discuss agentic AI workflows in pharmacology	Envisions AI agents automating modeling and simulation pipelines	Autonomous PK/PD modeling and analysis	[19]
14	Comparison of Different Machine Learning Methodologies for Predicting the Nonspecific Treatment Response in Placebo Controlled Major Depressive Disorder Clinical Trials	Original Research Article	Predict placebo response in MDD trials using ML	Gradient boosting improved response prediction over linear models	Placebo effect adjustment in trial design	[20]
15	Integrating Model‐Informed Drug Development With AI: A Synergistic Approach to Accelerating Pharmaceutical Innovation	Minireview	Combine AI with model‐informed drug development	Hybrid models improve efficiency and adaptability	AI‐enhanced dose optimization and simulation	[21]
16	First, Do No Harm: Addressing AI's Challenges With Out‐of‐Distribution Data in Medicine	Perspective	Discuss risks of applying AI to out‐of‐distribution data and methods to identify out‐of‐distribution before making predictions	Framework for detecting distribution shifts	Safe deployment of AI in variable clinical settings	[22]
17	Establishment and Validation of a Machine‐Learning Prediction Nomogram Based on Lymphocyte Subtyping for Intra‐Abdominal Candidiasis in Septic Patients	Original Research Article	Develop ML nomogram using lymphocyte subtyping	Improved early diagnosis accuracy	AI‐assisted infection risk prediction	[23]
18	Prediction of Cisplatin‐Induced Acute Kidney Injury Using an Interpretable Machine Learning Model and Electronic Medical Record Information	Original Research Article	Predict AKI risk in cisplatin patients	Interpretable ML improved clinical trust	EMR‐based toxicity risk screening	[24]
19	Augmented Intelligence in Precision Medicine: Transforming Clinical Decision‐Making With AI/ML and/or Quantitative Systems Pharmacology Models	Perspective	AI/QSP integration in precision medicine decisions	Case studies in dose tailoring	Personalized treatment planning	[25]
20	Comparing Machine Learning and Deep Learning Models to Predict Cognition Progression in Parkinson's Disease	Original Research	Compare ML and DL for predicting cognitive decline	DL showed marginal gains with more complex data	Early intervention planning in neurodegeneration	[26]
21	Practical Guide to SHAP Analysis: Explaining Supervised Machine Learning Model Predictions in Drug Development	Tutorial	Explain SHAP for ML interpretability	Demonstrates feature impact explanations	Transparent biomarker‐driven modeling	[27]
22	Hierarchical Deep Compartment Modeling: A Workflow to Leverage Machine Learning and Bayesian Inference for Hierarchical Pharmacometric Modeling	Tutorial	Combine ML with Bayesian hierarchical PK modeling	Hybrid modeling improved parameter estimation	Scalable complex PK modeling	[28]
23	Pharmacogenomic Augmented Machine Learning in Electronic Health Record Alerts: A Health System‐Wide Usability Survey of Clinicians	Original Research Article	Assess usability of pharmacogenomic AI alerts	Clinician feedback highlighted integration needs	Real‐time AI clinical decision support	[29]
24	Computational Drug Discovery Pipelines Identify NAMPT as a Therapeutic Target in Neuroendocrine Prostate Cancer	Original Research Article	AI pipeline identifies NAMPT as target	Validated computationally and experimentally	Target discovery in oncology	[30]
25	Explainable Machine Learning Prediction of Edema Adverse Events in Patients Treated With Tepotinib	Original Research Article	Predict edema risk in tepotinib patients	Explainability improved clinician adoption	AE risk mitigation in oncology	[31]
26	From Organs to Algorithms: Redefining Cancer Classification in the Age of Artificial Intelligence	Perspective	AI‐driven molecular reclassification of cancer	Proposes moving beyond tissue‐based taxonomy	Molecular subtype‐based trial design	[32]
27	Increasing Acceptance of AI‐Generated Digital Twins Through Clinical Trial Applications	Perspective	Digital twin adoption in trials	Strategies for clinician and regulator trust	In silico patient simulations	[33]
28	Accelerating Healthcare Innovation: The Role of Artificial Intelligence and Digital Health Technologies in Critical Path Institute's Public‐Private Partnerships	Perspective	AI/DHT integration in public–private partnerships	Examples from regulatory science initiatives	Accelerated regulatory submissions	[34]
29	Potential Meets Practicality: AI's Current Impact on the Evidence Generation and Synthesis Pipeline in Health Economics	Perspective	Evaluate AI's role in evidence synthesis for health economics	Showcases NLP and ML for literature review and meta‐analysis acceleration	Automating systematic reviews and cost‐effectiveness analysis	[35]
30	Rapid Identification and Phenotyping of Nonalcoholic Fatty Liver Disease Patients Using a Machine‐Based Approach in Diverse Healthcare Systems	Original Research Article	Identify NAFLD patients across health systems	Validated approach in diverse EHR datasets	Automated disease phenotyping from RWD	[36]
31	Real‐World Evidence in the Cloud: Tutorial on Developing an End‐To‐End Data and Analytics Pipeline Using Amazon Web Services resources	Tutorial	Build cloud pipeline for real‐world data analysis	Demonstrates scalable AWS architecture	Cloud‐native pharmacovigilance and RWE generation	[37]

In this piece, we aggregate contributions from over 30 manuscripts in this special issue, organized into three general categories (Figure 1, Table S1): Discovery and Preclinical Innovation, Clinical Development and Precision Medicine, and Postmarketing, Safety, and Real‐World Implementation. We also discuss the cross‐cutting aspects of regulation, ethics, and operations that may ensure AI achieves its potential. As readers progress through this editorial, they will see that the contributions in this issue do more than showcase technical innovation. They reflect a growing maturity in how the clinical pharmacology and translational science communities approach AI, which is not being expressed as a ‘one‐size‐fits‐all’ solution but rather as a toolkit, where the impact of AI will depend on thoughtful integration, appropriate validation, and a deeper understanding of both its strengths and limitations.

**FIGURE 1:**
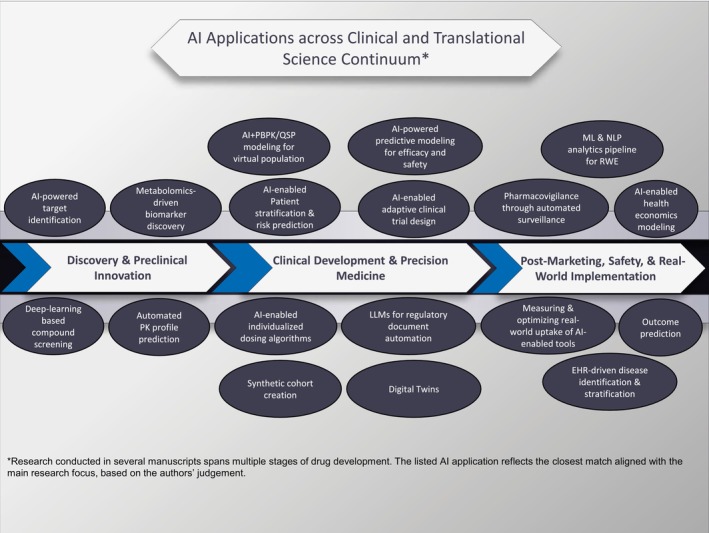
Applications of artificial intelligence across the *clinical and translational science* continuum, spanning discovery and preclinical innovation, clinical development and precision medicine, and postmarketing safety and real‐world implementation.

## 1 AI in Early‐Stage Drug Discovery and Preclinical Innovation

At the beginning of the drug development continuum—where drug developers identify targets, discover biomarkers, and create preclinical models—AI's intuitive ability to assess and map the meaning within massive, multidimensional datasets is, more often than not, beginning to be leveraged. At the earliest stages of discovery science, AI's transformative property is not necessarily its computational speed; rather, it is AI's ability to spot deep and subtle patterns that statistical approaches have most often failed to recognize, especially nonlinear relationships. This special issue includes many contributions that demonstrate how AI can now enable broader hypothesis generation, more rapid compound optimization, and more biologically founded predictions.

One area that is rapidly progressing is in silico pharmacokinetic (PK) and pharmacodynamic (PD) modeling. Walter et al., make a systematic examination of ML‐based approaches to empirical, compartmental, and physiologically based pharmacokinetic (PBPK) models for predicting plasma PK profiles in rats [9]. In a study with over 1000 small molecules, the authors conclude that mechanistic/compartmental PK models with the ML model, and direct ML‐based profile prediction both yield similar performance, while they all perform better than simple noncompartmental approaches (e.g., NCA‐based). Importantly, ML‐based approaches allow compounds to be prioritized and triaged earlier in the discovery pipeline based on predicted PK properties prior to synthesis or animal study, which speeds lead selection and reduces superfluous in vivo studies. Likewise, Pillai et al., introduce a pipeline for predicting plasma PK profiles in preclinical species (rats) from chemical structure with a chain of ML models [12]. Initially, their method uses molecular descriptors and ML to approximately predict in vivo clearance and volume of distribution, and uses the results to then train a second ML model to estimate the full plasma concentration‐time curve. Their platform is fairly accurate, especially for similar compounds to the training dataset (Tanimoto value > 0.5), with mean absolute percentage errors of approximately 150% most often, and it allows the investigator to have PK data even before any experimental data at all, which will further extend the role of AI in virtual screening and compound selection. These examples imply that AI can accelerate knowledge accumulation early in the input stage of discovery, so when questions arise during drug screening, there is much less to lose in terms of financial failure or opportunity to pivot.

In addition to these interesting research examples, the article by Zhang et al. represents an example of AI‐driven target identification by using a pipeline to develop a drug response prediction model of neuroendocrine prostate cancer [30]. This pipeline includes transcriptomics, high‐throughput drug screen (HTDS), and causal feature selection methods to derive new actionable targets—in this example identifying nicotinamide phosphoribosyltransferase (NAMPT) as a new target. Their research also presents experimental data with enhanced efficacy of NAMPT inhibitors, in NEPC models, and identifies potential predictive biomarker signatures for clinical translation. This study illustrates nicely how AI via omics data integration and in vitro validation can streamline target identification and further precision oncology approaches even as AI comes into the clinic. AI's ability to empower phenotype‐driven discovery is exemplified by Basile et al. [36], who developed and tested a rules‐based, machine‐driven approach for expedited and high‐fidelity identification and phenotyping of patients with nonalcoholic fatty liver disease (NAFLD) across multiple health systems. The application of large‐scale EHR data in their work yielded high positive predictive value, and high levels of sensitivity toward NAFLD case‐finding and risk stratification, illustrating real‐world translational viability of ML for discovery directly in patient populations. Furthermore, the algorithm also permits enrollment into focus interventions while sustaining risk stratification relevant toward experimental or therapeutic development, reflecting the emergence of insight from population‐level EHR mining as much as laboratory molecular assays.

From a generative modeling standpoint, Titar and Ramanathan present variational autoencoder (VAE)–based generative modeling in order to simulate populations with renal, hepatic, metabolic, and cardiac disease with respect to multidimensional physiological biomarkers that are directly applicable for dose decision‐making [11]. Their work shows that tabular VAE models can sufficiently approximate distributions of continuous physiological determinants of drug dosing; however, performance for categorical, and particularly rare disease states, is constrained. This application of generative AI fosters simulation‐based hypothesis generation and clinical trial design focused on real‐world interindividual variability, without reliance on either costly or hard‐to‐obtain data. In connecting mechanistic and data‐driven paradigms, Elmokadem et al. propose, and demonstrate, a research workflow for hierarchical deep compartment modeling (HDCM) [28]. In the proposed framework, neural networks are embedded in compartmental PK model structure and uncertainty and variability are handled with Bayesian inference. The HDCM framework allows for scalable modeling of population‐level trends and individual‐level heterogeneity; consequently, the method includes utility (like SHAP analysis) for making predictions based on model input (covariates visible in the model). Notably, this hybrid modeling permits modeling beyond previous limitations, where ML generally provides no estimates of uncertainty nor mechanistic extrapolation capability, so the method should allow broader applicability for both simulation and interpretation in preclinical pharmacology.

Collectively, all these studies indicate that AI in the early stages of discovery has progressed well past retrospective pattern mining and is focused on predictive and generative modeling, which contribute directly to experimental methods and aid resource allocation, and ultimately embedding enhanced efficiency, elasticity, and patient pertinence for downstream clinical development.

## 2 AI in Clinical Development: Transforming Trials and Personalizing Treatment

Once you have a candidate molecule, the center of gravity shifts from bench to clinic, and uncertainty is driven by human heterogeneity, not chemistry. Clinical development, which can represent the most resource‐demanding phase of drug development, has historically been characterized by long timelines, rigid protocols, and minimal flexibility once the trial is initiated. Artificial intelligence is questioning this framework by shifting to a more spontaneous and data‐driven approach to the design of trials, patient selection, and personalization of treatment. In each of the studies in this special issue, AI was employed in three interrelated ways: optimizing trial design and execution, refining patient stratification and predictive modeling, and personalizing treatment and dose optimization.

### 2.1 Optimizing Trial Design and Execution

The fast‐paced growth of AI in trial design and operational execution was a salient topic across the discussions during the ASCPT 2024 AI Preconference, and evidenced in the summary by Shahin et al. [8] Their summary illustrates how AI is providing opportunities for innovations like generative models for generating study protocols, digital health technologies used for endpoint surveillance, and the use of explainable AI in dose‐finding and covariate analysis. In contrast to the summary focusing on adaptive randomization and specifications of protocol simulation, the summary paints a broad view of AI‐enabled innovations that are now underway in modern drug development. Shahin et al. highlight the importance of multidisciplinary partnerships as AI becomes implemented in regulatory submissions and operational decision‐making. They emphasize that engaging specialists from statisticians and social scientists to regulators is critical, and they insist on consideration for scientific rigor and methodological transparency when embracing new computational tools.

Continuing from the operational utility of AI, Huh et al. investigated using Meta Llama 3, an open‐source large language model, for automating the systematic identification and characterization of clinical trials with decentralized components from registry databases [18]. Instead of offering a mechanism for regulatory review and approval of the protocol, this study demonstrated how large language models could systematically sort and organize trials by decentralized components based on the free text description of the trials. They conclude that models like these have considerable value for trend analytics and extracting structured data from unstructured data sources, but they also warn that it is sensitive to the models the user gets transparency and accountability outputs. Their work is best understood as a proof‐of‐concept for the use of AI to support the operational review of decentralized clinical trials, and shortcomings remain because of changing registries' terminology and scope.

Regarding data operations transformation, Podichetty et al. outline the emerging end‐to‐end analytic pipeline work at Critical Path Institute (C‐Path) within the Amazon Web Services (AWS) cloud [34]. Their example is less a case study by itself, but, more of an overview for ongoing transformation where traditional, static data pipelines are replaced by AI‐augmented, cloud‐native infrastructures. They characterize these future platforms as scalable, interoperable infrastructures that enable rapid assembly, validation and surveillance of real‐world data for regulatory‐grade evidence generation. Near‐real‐time data movement and data synthesis are the current state, as they note, and this is part of the iterative and collaborative nature of trial data management in modern R&D.

### 2.2 Refining Patient Stratification and Predictive Modeling

Stepping into areas of patient stratification and predictive modeling, the papers of this special issue highlight the power of AI to work with high‐dimensional clinical and biological data to provide risk estimates and tailor precision medicine. Zhang et al. explain the usage of ML to develop and validate a nomogram which utilized lymphocyte subtyping and significantly increased predictive precision for intra‐abdominal candidiasis in sepsis patients [23]. Ambe et al. leverage interpretable ML models utilized on electronic medical records to predict acute kidney injury from cisplatin‐based chemotherapy highlighting that model transparency and early predictions were critical for practical use, starting before chemotherapy [24]. Lan et al. introduce MoLPre (Machine Learning Model for Lung Cancer Metastasis Prediction), a ML‐based platform to predict metastasis in early‐stage lung cancer creating new possibilities in personalized therapy approaches during the surgical decision [16]. Amato et al. utilize explainable AI to analyze data from tepotinib trials, leading to the idea that safety signals—like edema—could be included for precision treatments not just in postmarketing but during clinical developments [31].

In agreement with those advancements, Khozin's “From Organs to Algorithms” proposes transforming oncology classification frameworks from anatomical overclassification to an AI‐enabled, data‐driven, molecular‐based stratification [32]. He offers a vision where the future of oncology will be increasingly shaped by multiomics inputs and computational profiling, wherever boundary disease entities are defined and patients are chosen for therapeutics. As a whole, these papers signal a change from static risk scores and empiric registries to fully integrated and transparent decision‐support systems that leverage algorithms. They signal that AI‐supported systems would do the most good and be the most useful when both the logic and data sources are open and clear to clinicians and end users. This contributes to transparency, regulatory pathways, and user‐accepted evidence across fields.

### 2.3 Personalizing Treatment and Dose Optimization

In the AI era, personalizing treatment is not simply about identifying the right patient, but also about tailoring the right intervention. In this issue, Venkatapurapu et al. addressed the role of augmented intelligence, that is, a hybrid of AI/ML methods and quantitative systems pharmacology (QSP) models, in precision medicine [25]. They share the opportunities that clinical decision support systems utilizing a predictive model, digital twin, or both framed on both AI and QSP models give an advantage that can be continuously mapped onto the changing patient journey. The authors present digital twin‐enabled chronic disease management platforms and discuss how hybrid QSP‐ML models can facilitate shared decision‐making and provide scenario planning for dose optimization with an overall consideration for data privacy, explainability, and trust, which are pausing points in clinical care AI adoption. Similarly, Raman et al. continue this discussion on model‐informed drug development (MIDD) and AI together to amplify pharmaceutical development [21]. Their minireview discusses the possibilities created when mechanistic models (traditional MIDD) and cutting‐edge AI/ML are combined, focusing on how these approaches could streamline patient selection, define trial endpoints, and predict drug response. They demonstrate how a combined approach can be implemented through concrete examples spanning early discovery to clinical trials, with emphasis on what model interpretability, regulatory buy‐in, and a collaborative, interdisciplinary training environment will need to leverage model‐based personalization in medicine.

Another newer approach to individualization is using AI‐derived digital twins, virtual patients that integrate rich, heterogeneous, and real‐time data. Assigning a computational infrastructure that tracks every parameter within, or as close to the point of, care can provide knowledge in the management of chronic diseases at the clinical site. Vidovszky et al. specifically address clinician and patient acceptance of AI‐driven digital twins in clinical trial contexts [33]. They define digital twins as AI models potentially capable of reflecting individualized treatment outcomes, and their points of use in trial optimization are discussed (e.g., virtual control arms, covariate adjustment, patient‐specific predictions). The authors argue to utilize regulated clinical trial settings to build trust in digital twin tools, and suggest actionable explainability, rigorous validation (SHAP and LIME), stakeholder engagement at the outset, and regulatory clarity as adoption facilitators.

Pharmacogenomics remains an important lever for individualizing therapy, particularly facilitated through AI in clinical decision support. Grant et al., share a system‐wide survey of clinician perceptions of pharmacogenomic alerts enhanced by AI in an electronic health record (EHR) system [29]. The findings showed strong interest, but skepticism: clinicians are interested in patient‐specific actionable alerts, and those alerts should be respectful of clinician workflow. The finding emphasized the overall theme that for AI to be successful, there is a complementary requisite of technical rigor and human‐centered design aligned to clinical workflows.

Supporting these scientific studies, are methodological tutorials that provide commonsense foundational enablers for AI transparency and infrastructure. Ponce‐Bobadilla et al., deliver a practical guide to SHapley Additive exPlanations focused on the interpretability of supervised machine learning in drug development [27]. The tutorial examples work through examples of how SHAP might be used to understand feature contributions across ML model types—regression, classification, and time series prediction—and lower a long‐standing barrier to AI/ML efficiencies in biomedicine. Additionally, Wiens et al., provide a tutorial on XGBoost for clinical and translational scientists, demonstrating the implementation and optimization of the popular ensemble modeling algorithm using clinical trial‐type datasets in R [17]. The tutorial's use‐case‐driven and thorough R code walkthroughs offer practical and actionable pathways for particularly statisticians and pharmacometricians interested in applying ML, to real‐world data. Finally Anderson et al., describe a cloud‐based, end‐to‐end, RWE analytics pipeline based on Amazon Web Services [37]. Their tutorial—the only tutorial in this issue that tells the story of underlying digital health infrastructure—details scalable data ingestion, transformation, visualization, and machine learning deployment (with relevant examples through the CURE ID platform). Taken with the generative dosing model frames of reference in the issue, this type of process coupled with next‐generation platform tools demonstrates how to overcome technical barriers with the mindset of democratizing advanced analytics and AI for trial operations, dose optimization, and dataset integration.

## 3 AI in Postmarketing Surveillance, Real‐World Evidence, and Pharmacovigilance

Although AI has received extensive coverage and advancement for drug development, accelerating drug discovery and optimizing clinical trial conduct is important; its influence does not stop at the approval stage of development. The postmarketing hurdles start as we shift from proving effectiveness in a controlled environment, to understanding the safety, efficacy, and uptake of new therapeutic interventions in the unpredictable and heterogeneous reality of clinical practice, in a way that recognizes the complexities of human decision making and the impact of drug use on patient health. AI‐enabled pharmacovigilance and real‐world evidence (RWE) creation also allow faster identification of safety signals in a time and place when new therapies are taken up by the healthcare system, while providing the ability to detect rare and less common adverse events that may not have been identified prior to approval through clinical trial processes, and supporting identification of nuanced patterns of benefit and risk in different patient subgroup populations.

This issue highlights a number of studies that illustrate the ability of AI to move postmarket monitoring from a passive and retrospective exercise to a proactive and dynamic process. The important contributions showcased in this issue exemplify how applying ML approaches—using electronic health records (EHR)—to detect clinically relevant phenotypes and adverse events at scale can be accomplished. For example, AI methods to phenotype patients with nonalcoholic fatty liver disease (NAFLD) were demonstrated to rapidly detect individuals with the disease across multisystem healthcare applications, by combining medical diagnostic codes, laboratory values, and imaging reports for a scalable surveillance process [36]. Methods for identifying phenotypes in a scalable capacity are not purely academic, but rather enhance our ability to increase efficiency in observational study recruitment, engage with stratified safety monitoring, and target therapeutic interventions to subpopulations who need them most.

Another critical enabler of postmarket AI is leveraging cloud‐native analyses pipelines that allow for the ingestion and harmonization of a multitude of disparate data sources over time. One of the tutorials in the issue outlined a method to develop a fully integrated RWE pipeline based on AWS, to securely and reproducibly ingest, process, and analyze large‐scale clinical datasets [37]. Such architectures for pharmacovigilance are necessary to enable scale beyond local, siloed systems, while supporting opportunities for global collaboration where compliance, privacy, and performance are critical.

Detecting adverse events, especially rare and subtle ones, remains a central focus of AI in pharmacovigilance. The studies in this issue demonstrate how explainable AI models that used clinical, laboratory, and treatment information to predict drug‐induced adverse events (edema for patients treated with tepotinib) provide outputs that the clinician could interpret, validate, and trust [31]. Models predicting cisplatin‐induced acute kidney injury with EMR data similarly demonstrated not just high accuracy, but also reason to promote the use of transparency of the model to inform clinical decision‐making [24].

The digital twin concepts—AI produced and generated patient‐specific virtual representation—emerged in this issue as a value‐added pathway between the simulation of the premarket simulations, and the personalization of the postmarket experience [33]. The increased acceptance of digitally twins that are AI‐produced for clinical trial applications as discussed in this issue, opens possibilities for their extension into real‐world monitoring with the ability to simulate treatment trajectories under alternative scenarios to inform safety and efficacy before, or at the point of therapy, for patients with healthcare needs. By integrating these approaches, EHR phenotyping, cloud‐native analytics, explainable adverse event models, and digital twins, the postmarket phase can evolve into a continuous learning system. AI does not replace pharmacovigilance expertise; rather, it enhances the ability to detect, contextualize, and act upon new information in a way that is both faster and more precise than traditional methods.

## 4 Regulatory, Ethical, and Implementation Considerations

The use of AI in drug development will encounter regulatory, ethical, and implementation challenges as it moves through the drug development continuum. While AI has been proven to work in theory, it will be important to validate and regulate these systems in a way that creates trust from all parties (regulators, clinicians, and patients). For example, regulatory perspectives are changing rapidly. The FDA, EMA, and other agencies have issued guidance on AI systems being used in medical devices (including software), and drug development, which involves interpreting AI in terms of transparency, reproducibility, and robustness [38, 39, 40, 41, 42]. Some articles in this special issue deal with these principles directly. For example, the included discussions on the problems with out‐of‐distribution data in medicine highlight the importance of rigorous validation across diverse groups of patients to prevent any degradation in performance in situations in which AI models encounter new unknown data inputs [22].

There was a congruence of messages about explainability and interpretability being fundamentally required for clinical implementation. Although the AI model may be a high‐performing “black box,” so long as it achieves a benchmark, it is not going to gain regulatory and clinical acceptance because we do not know why it made a prediction. There are tutorials on AI tools like SHAP (SHapley Additive exPlanations), which are included in this issue, and we believe this is a way to implement interpretability into your model development analysis pipelines [27]. This will help during the regulatory step, but also helps trusted clinicians work in a high‐consequence environment that involves dose selection, adverse event prediction and diagnostic classification. Additionally, bias and equity remain important issues from an ethical standpoint. AI systems that are trained on nonrepresentative datasets can consistently reinforce or exacerbate health care disparities. Research published in this special issue that takes advantage of multisite EHR, federated learning methods, and/or synthetic data generation with variational autoencoders presents methods of diversifying datasets for training, and balancing privacy for the patient. These approaches are important to ensure AI‐based recommendations do not regularly discriminate against underrepresented populations.

Notwithstanding, realities of implementation must be embraced. Multiple successful AI models are stuck in an academic paper because of the challenges embedding those models into clinical and research workflows. The implementation of AI‐based applications in complex clinical environments requires a strong data governance framework, must be able to integrate with current health IT workflows, and must place the user at the center to ensure the AI output is useful, timely, and actionable to the end users. The case studies found in this special issue—including integrating pharmacogenomic alerts into a clinical EHR‐sourced workflow [29], and designing AI‐assisted platforms for implementing and executing trials [37]—illustrate that technical excellence does not stand alone, but must incorporate intentionality around workflow to achieve meaningful impact.

Finally, the potential to collaborate with multiple stakeholders is paramount. AI is a timely development in clinical pharmacology and translational science, and occupies space at the interface of computational science, clinical research, regulatory policy, and advocacy for patient rights and patient‐centeredness. Studies in this special issue illustrate opportunities for partnerships between academia and industry, between regulators and technology providers, not just to enhance methodological development but also to define common measurements, pathways to validation, and paths to scaling.

## 5 Conclusion

The published manuscripts in this special issue follow an unbroken analytical thread from molecular hypothesis to real‐world impact. AI‐enabled learning chemistries and hybrid physiological, biological and chemical modeling are a clear trajectory to digital twin trial engines, toxicity monitors at the bedside, and dashboards of safety at a population level; all enabled by an AI agenda that balances scientific rigor with operational reality. Collectively these manuscripts reposition AI not as a toolkit to accompany the practices of contemporary clinical pharmacology, but rather as the connective tissue of a new, postpandemic pharmacology. However, in order to realize full potential, transparency, graduated validation, and equity must be valued in every algorithm, every dataset, and every decision.

## Disclosure

This publication reflects the views of the authors and should not be construed to represent the FDA's views or policies.

## Conflicts of Interest

The authors declare no conflicts of interest.

## Supporting information


**Data S1:** cts70383‐sup‐0001‐supinfo.docx.
