# Supplementary material for: Artificial Intelligence in Clinical and Translational Science: From Bench Insights to Bedside Impact
Source: Clin Transl Sci. 2025 Nov 3;18(11):e70383. doi: 10.1111/cts.70383 (PMC12580936; doi:10.1111/cts.70383)
Supplement: Supplementary file 1 — Data S1: cts70383‐sup‐0001‐supinfo.docx. [file CTS-18-e70383-s001.docx]

**SUPPLEMENTAL MATERIALS**

**Artificial Intelligence in Clinical and Translational Science: From Bench Insights to Bedside Impact**

Mohamed H. Shahin^1^ and Qi Liu^2^

1 Pfizer Research & Development, Groton, Connecticut, USA

2 Office of Clinical Pharmacology, Office of Translational Sciences, Center for Drug Evaluation and Research, U.S. Food and Drug Administration, Maryland, USA

Disclaimer:

This publication reflects the views of the authors and should not be construed to represent the FDA’s views or policies.

**Table S1. Summary of manuscripts in the Clinical and Translational Science AI-Themed Issue**

| **#** | **Title** | **Drug Development Stage*** | **Objective** | **Key Highlights** | **Potential AI Application** | **Ref** |
| --- | --- | --- | --- | --- | --- | --- |
| 1 | Predicting Pharmacokinetics in Rats Using Machine Learning: A Comparative Study Between Empirical, Compartmental, and PBPK-Based Approaches | Discovery/Preclinical Innovation | Systematically comparing ML-based approaches with empirical, compartmental, and PBPK models for predicting pharmacokinetic profiles in rats following intravenous dosing, aiming to enhance preclinical candidate selection | Three ML-integrated methods—Pure-ML, Compartmental-ML, and PBPK-ML—outperformed baseline approaches by accurately predicting full plasma concentration-time profiles, offering a practical framework for early drug ranking and dose estimation; the analysis covers around 8,000 compounds and assesses model bias and accuracy across timepoints | Machine learning combined with mechanistic PK modeling enables automated high-throughput prediction of complete PK profiles from chemical structures, facilitating rapid prioritization of compounds for drug discovery and improving translatability to human dose projections | ^1^ |
| 2 | Large Language Models and Their Applications in Drug Discovery and Development: A Primer | Discovery/Preclinical Innovation | Provides a comprehensive primer on large language models (LLMs), focusing on fundamental concepts and emerging applications in drug discovery and development, including use cases in clinical pharmacology and translational science. | LLMs are transforming workflows in target identification, compound synthesis, safety assessment, evidence generation, and medical writing; practical examples illustrate advancements in regulatory intelligence (RIA), automation of medical writing (REGAIN), and intelligent agent frameworks in quantitative clinical pharmacology (Apollo-AI). | LLMs, such as GPT-4 and domain-specific models, can automate literature mining, accelerate systematic reviews and model-based meta-analyses, generate analysis code for PK/PD modeling, streamline regulatory document preparation, and enable digital twin simulation using real-world data, with human oversight recommended for validation. | ^2^ |
| 3 | AI In Action: Redefining Drug Discovery and Development | Discovery/Preclinical Innovation | Provides insights into AI-driven advances across drug discovery, translational research, and development, summarizing clinical pharmacology community survey results and case studies to highlight practical impacts, successes, and directions for AI integration in pharmaceutical R&D | AI models—such as AlphaFold and PharmBERT—enable rapid protein structure prediction, target/adverse-event associations, and information extraction from drug labels; AI use has led to higher phase I success rates for candidate drugs, and generative AI tools are increasingly impacting regulatory writing, pharmacometrics model selection, and efficient clinical trial operations | AI methods—including deep learning, conventional ML, and generative large language models—can accelerate molecule design, automate regulatory document drafting, improve model-based analyses, and enable precise, data-driven decision-making throughout drug discovery, development, and regulatory submission processes | ^3^ |
| 4 | Variational autoencoders for generative modeling of drug dosing determinants in renal, hepatic, metabolic, and cardiac disease states | Discovery/Preclinical Innovation | Evaluates variational autoencoders (VAE) for generative modeling of physiological determinants of drug dosing (PDODD) in renal, hepatic, metabolic, and cardiac disease states, and assesses whether VAE can produce synthetic patient populations for simulation and pharmacometric analyses | A tabular VAE accurately captured high-dimensional joint distributions and clinical biomarker patterns in NHANES data, generating realistic virtual patient populations for continuous biomarkers but with only moderate fidelity for rare binary variables; graphical, statistical, and multidimensional projection methods support the VAE's ability to encode disease-related changes in dosing determinants | VAE-based models enable virtual population creation for clinical trial simulation, high-fidelity disease state representation with patient-level dosing biomarkers, and synthetic data generation for scenarios with limited real patient data, supporting precision pharmacometrics and in silico trial design | ^4^ |
| 5 | Machine learning framework to predict pharmacokinetic profile of small molecule drugs based on chemical structure | Discovery/Preclinical Innovation | Presents a machine learning framework that predicts the plasma pharmacokinetic (PK) profile of small molecule drugs directly from chemical structure, aiming to reduce reliance on animal experiments and accelerate early compound screening | The model predicts in vivo clearance and volume of distribution from SMILES molecular representations, then uses these as inputs to ML algorithms (e.g., XGBoost) to estimate PK profiles; results show reasonable accuracy versus observed PK data, with error metrics around two-fold for most compounds, and performance comparable to PBPK models | ML-driven PK profile prediction enables virtual preclinical PK assessment, rapid compound prioritization, and mechanistic modeling integration in drug discovery—offering scalable, animal-sparing alternatives for early-phase candidate evaluation and design optimization | ^5^ |
| 6 | Non‐targeted metabolomics for the identification of plasma metabolites associated with organic anion transporting polypeptide 1B1 function | Discovery/Preclinical Innovation | Aimed to discover robust endogenous plasma biomarkers of OATP1B1 function using a hypothesis-free, non-targeted metabolomics approach combined with linear regression and machine learning in healthy volunteers | Among 9,152 metabolite features, the study confirmed GCDCA-3G and GDCA-3G as sensitive, specific OATP1B1 biomarkers and identified several additional candidates (LPE 22:5, pregnanolone sulfate, pregnenolone sulfate); random forest and gradient boosting decision trees successfully stratified subjects by OATP1B1 genotype/function | Machine learning (random forests, GBDT) can be combined with non-targeted metabolomics to automate biomarker discovery, stratify transporter activity, and support drug–drug interaction risk assessment and personalized medicine in clinical pharmacology. | ^6^ |
| 7 | Machine learning identifies fatigue as a key symptom of fibromyalgia reflected in tyrosine, purine, pyrimidine, and glutaminergic metabolism | Discovery/Preclinical Innovation | Uses machine learning on clinical and metabolomic data to identify fatigue as the dominant symptom of fibromyalgia, and links this symptom to distinct metabolic pathway changes, particularly under metabolic and physical stress. | Fatigue alone distinguished fibromyalgia patients from controls with 86% accuracy, while ML identified tyrosine, purine, pyrimidine, and glutaminergic pathway alterations; 13 key metabolites and stress-induced metabolic shifts were reliably associated with symptom burden and pathophysiological processes | AI/ML methods can automate multidimensional integration of symptom and metabolite data to derive diagnostic biomarkers, stratify patients, and delineate metabolic phenotypes for individualized therapy development in fibromyalgia and related chronic pain disorders | ^7^ |
| 8 | Leveraging In Silico and Artificial Intelligence Models to Advance Drug Disposition and Response Predictions Across the Lifespan | Clinical Development/Precision Medicine | Review and discuss how in silico and artificial intelligence models can advance the prediction of drug disposition and response across diverse populations and life stages in drug development | Summarizes applications of PBPK and QSP/QST modeling for virtual populations, presents a case study in drug-induced liver injury for postmenopausal women, and discusses strategies and challenges for integrating AI with modeling to improve drug development and dosing predictions. | AI methods, such as machine learning and generative adversarial networks, are highlighted for analyzing real-world data, creating virtual populations, and identifying physiological determinants to enhance drug exposure and outcome predictions, particularly for underrepresented patient groups | ^8^ |
| 9 | AI-Driven Applications in Clinical Pharmacology and Translational Science: Insights From the ASCPT 2024 AI Preconference | Clinical Development/Precision Medicine | Summarizes AI-driven applications in clinical pharmacology and translational science, highlighting insights from the ASCPT 2024 AI preconference focused on advancing drug development, clinical trials, and patient care using AI and ML | Covers the rapid integration of AI—including generative models, explainable ML, and large language models—across the drug development lifecycle, dose optimization, digital biomarkers, regulatory submissions, and patient monitoring, demonstrating applications in digital endpoint assessment, synthetic patient data generation, and immune digital twins; ethical and regulatory considerations and future trends are discussed | AI enables automated regulatory document drafting, digital biomarker and endpoint development, explainable risk prediction for adverse events, enrichment of clinical trials using generative adversarial networks, and construction of biomedical knowledge graphs and conversational frameworks to democratize data analysis and decision-making in precision medicine | ^9^ |
| 10 | MoLPre: A Machine Learning Model to Predict Metastasis of cT1 Solid Lung Cancer | Clinical Development/Precision Medicine | Develops and validates MoLPre, a machine learning model using clinicopathological features to predict the risk of metastasis in patients with early-stage (cT1) solid lung cancer for improved therapeutic planning and risk stratification | A random forest-based classifier incorporating nine clinical factors achieved high predictive accuracy (AUC 0.92, precision 0.93) in a single-center cohort, and the model is deployed as an open-access web tool for clinicians to estimate metastatic risk non-invasively using routinely collected data. | Enables rapid, automated risk prediction for lung cancer metastasis at point-of-care, supporting clinical decision-making and potentially triaging patients for treatment intensity or surveillance using interpretable machine learning algorithms | ^10^ |
| 11 | A Tutorial and Use Case Example of the eXtreme Gradient Boosting (XGBoost) Artificial Intelligence Algorithm for Drug Development Applications | Clinical Development/Precision Medicine | Provides a practical tutorial on the eXtreme Gradient Boosting (XGBoost) AI algorithm, focusing on foundational concepts and hands-on code for regression and classification problems in drug development using open-source clinical trial-like data | The tutorial details the implementation of XGBoost in R, explains model structure and optimization, includes working examples with breast cancer and liver disorder datasets, and emphasizes model evaluation, hyperparameter tuning, and application caveats for clinical research | XGBoost can be used in drug development to predict clinical outcomes, risk or response, support therapeutic drug monitoring, enhance pharmacokinetic profile prediction, and handle large, complex datasets typical of real-world evidence and clinical trial data | ^11^ |
| 12 | Exploration of Using an Open-Source Large Language Model for Analyzing Trial Information: A Case Study of Clinical Trials With Decentralized Elements | Clinical Development/Precision Medicine | Explores the feasibility and performance of open-source large language models (LLMs), particularly Llama 3, for analyzing trends and decentralized elements in clinical trial registries using unstructured text data | Fine-tuned LLMs demonstrated improved sensitivity and computational efficiency in identifying decentralized clinical trials, with the larger 70B-parameter model excelling at extracting specific decentralized elements; annual DCT numbers increased notably after COVID-19, and phase 2 studies became most prevalent from 2020 onward | LLMs can automate and scale the mining of large-scale free-text clinical trial registries, enabling rapid identification of study designs and elements that are not machine-readable, thereby supporting trend analyses and registry-based research in clinical pharmacology and translational science | ^12^ |
| 13 | Agents for Change: Artificial Intelligent Workflows for Quantitative Clinical Pharmacology and Translational Sciences | Clinical Development/Precision Medicine | Provides a comprehensive review of agentic workflows powered by AI agents for quantitative clinical pharmacology and translational sciences, detailing how modular, collaborative AI agents can streamline data analysis, modeling, and decision-making in drug development and clinical research | Agentic workflows integrate specialized AI agents (including LLMs), domain expertise, and existing analytic tools to automate routine tasks, optimize pharmacokinetic and pharmacodynamic modeling, and support reproducible, privacy-compliant clinical trial analyses; the manuscript presents practical examples such as InsightRX Apollo-AI and BioChatter Reflexion Agent, and addresses challenges around integration, reproducibility, benchmarking, and regulatory compliance | Modular AI agents within agentic workflows can automate PK/PD analyses, assist with clinical trial design, aggregate and interpret biomedical knowledge graphs, generate reports, and bridge the gap between human and machine expertise, while supporting data provenance, benchmarking, and regulatory workflow requirements for transparent, scalable, and fair decision-making in drug development and personalized medicine | ^13^ |
| 14 | Comparison of Different Machine Learning Methodologies for Predicting the Non‐Specific Treatment Response in Placebo Controlled Major Depressive Disorder Clinical Trials | Clinical Development/Precision Medicine | Compares several machine learning methodologies—including ANN, gradient boosting, random forest, k-nearest neighbors, lasso, SVM, and logistic regression—for estimating the individual probability of non-specific (placebo) treatment response in randomized, placebo-controlled major depressive disorder trials | Multilayer perceptron ANN models outperformed all other tested approaches for predicting placebo response, with cross-validation showing strong accuracy, sensitivity, and precision; ANN-based estimation of placebo responder probability enables greater effect size and signal detection by removing high-propensity placebo cases from analyses, supporting more reliable assessment of drug efficacy | ANN, random forest, and gradient boosting models can be prospectively integrated into trial analysis pipelines to identify, stratify, and adjust for placebo responders, enhancing effect size estimation and enabling enriched population selection for future randomized trials in depression and psychiatric drug development | ^14^ |
| 15 | Integrating Model‐Informed Drug Development With AI: A Synergistic Approach to Accelerating Pharmaceutical Innovation | Clinical Development/Precision Medicine | Reviews the integration of model-informed drug development (MIDD) and artificial intelligence (AI), highlighting their synergistic potential to accelerate pharmaceutical innovation, optimize drug candidate selection, dosage, and trial design, and enable personalized treatment strategies | Describes how AI—including machine learning, deep learning, and generative models—enhances every stage of drug development from target identification to clinical trial optimization; case studies illustrate the practical benefits in patient selection, endpoint definition, drug combination prioritization, and real-world impact, while challenges in data quality, model interpretability, and regulatory standards are addressed | AI-driven approaches (such as LLMs, ML, and GenAI) can unify diverse data sources for trial simulation, automate regulatory document drafting, support explainable biomarker and endpoint identification, streamline PK/PD modeling, and foster data-driven decision-making for drug development and regulatory submissions. | ^15^ |
| 16 | First, Do No Harm: Addressing AI's Challenges With Out-of-Distribution Data in Medicine | Clinical Development/Precision Medicine | Addresses the challenges posed by out-of-distribution (OOD) data for medical AI models, proposing prescreening strategies and OOD detection algorithms to avoid or flag unreliable AI predictions and promote safer, more responsible deployment in clinical settings. | OOD samples can arise from demographic or practice differences and lead to harmful medical decisions, but AI-enhanced OOD detection (using deep learning, anomaly scores, and unsupervised models) demonstrably improves model reliability; integrating OOD screening in workflows enhances safety, trust, and inclusivity and helps define scope of use for AI in medicine | OOD detection algorithms—such as autoencoders, nearest-neighbor deep classifiers, and ensemble models—can be embedded into clinical AI workflows to prescreen data for distributional mismatches, minimize risk, support trial cohort enrichment, and highlight gaps in data diversity for future training and validation | ^16^ |
| 17 | Establishment and Validation of a Machine-Learning Prediction Nomogram Based on Lymphocyte Subtyping for Intra-Abdominal Candidiasis in Septic Patients | Clinical Development/Precision Medicine | Develops and validates a machine learning-driven nomogram using lymphocyte subtyping and clinical risk factors for early, bedside prediction of intra-abdominal candidiasis (IAC) in septic patients, aiming to improve rapid risk stratification and timely initiation of antifungal therapy | A random forest model identified high-dose corticosteroid receipt, CD4/CD8 T-cell ratio, total parenteral nutrition, gastrointestinal perforation, BDG positivity, and broad-spectrum antibiotic receipt as independent predictors, culminating in a nomogram with superior predictive performance (AUC ~0.82) over conventional scoring systems and allowing stratification into low, moderate, and high risk groups for clinical decision support | The approach demonstrates how machine learning models can be used to automate risk scoring, enhance early AI-driven detection, and guide antifungal therapy for IAC in complex infectious disease settings, with potential extension to bedside clinical decision support and biomarker discovery through deeper immune data mining | ^17^ |
| 18 | Prediction of Cisplatin-Induced Acute Kidney Injury Using an Interpretable Machine Learning Model and Electronic Medical Record Information | Clinical Development/Precision Medicine | Develops and validates an interpretable machine learning model using electronic medical record data to predict the risk of cisplatin-induced acute kidney injury (Cis-AKI) within 14 days of chemotherapy initiation, aiming to enable early identification of high-risk patients | A CatBoost model with 29 clinical variables, including labs, medications, and dosing, achieved a ROC-AUC of 0.78; SHAP analysis identified intravenous magnesium use (protective) and loop diuretics (risk) as major predictors, supporting individualized monitoring and preventive strategies based on EMR-derived risk scoring | Demonstrates how interpretable AI can automate real-time risk prediction for drug-induced adverse events—allowing dynamic risk monitoring, clinical decision support, and deeper mechanistic insights for precision prevention of Cis-AKI in oncology patients. | ^18^ |
| 19 | Augmented intelligence in precision medicine: Transforming clinical decision-making with AI/ML and/or quantitative systems pharmacology models | Clinical Development/Precision Medicine | Examines how augmented intelligence—integrating AI/ML, clinical decision support systems, and quantitative systems pharmacology (QSP) models—transforms precision medicine by empowering clinicians with data-driven insights for personalized treatment and shared decision-making | Augmented intelligence platforms (digital twins, AIML-powered CDSS) personalize care by using real-time patient data and predictive modeling; case studies include epilepsy surgery, Crohn’s disease, diabetes nutrition planning, depression, and oncology, while also discussing workflow barriers, regulatory hurdles, and trust-building using interpretable and actionable insights | Clinical digital twin systems and AIML-powered CDSS can optimize treatment trajectory for individual patients, automate ongoing risk and outcome prediction, and provide physicians with decision augmentation—enabling patient-specific therapy, clinical trial enrichment, and proactive disease management in diverse medical domains | ^19^ |
| 20 | Comparing machine learning and deep learning models to predict cognition progression in Parkinson's disease | Clinical Development/Precision Medicine | Develops, validates, and compares sequential machine learning and deep learning models—including Markov, LSTM, and transformer networks—to predict cognitive progression (normal, MCI, dementia) over 1–3 years in Parkinson’s disease patients using longitudinal clinical data | The temporal fusion transformer (TFT) model consistently outperformed Markov and LSTM models, and an ensemble approach yielded the highest inverse probability weighted F1 scores for predicting rare transitions (MCI, dementia); the use of simple, noninvasive predictors enables broad clinical applicability for individual patient care planning | Sequential deep learning and ensemble modeling approaches (like TFT) facilitate accurate, multi-horizon clinical prediction of cognitive decline in neurodegenerative diseases, offering scalable tools for prognosis, personalized care trajectory, and therapeutic intervention optimization in Parkinson's disease and beyond | ^20^ |
| 21 | Practical guide to SHAP analysis: Explaining supervised machine learning model predictions in drug development | Clinical Development/Precision Medicine | Provides a practical guide to SHAP (Shapley Additive Explanations) analysis for interpreting supervised machine learning model predictions in drug development, illustrating its application across standard regression, classification, and time-series models | Details SHAP mathematics and properties, visualization plots, practical considerations for binary endpoints and time-dependent data, and shows worked code examples in Python and R for XGBoost, random forest, neural networks, and classical regression; limitations and model-agnostic extensions are discussed | SHAP analysis enables global and local explanation of ML predictions for ADME, PK/PD, exposure-response, patient stratification, biomarker identification, and real-world clinical decision support, enhancing trust, transparency, and regulatory acceptance for black-box AI models in drug development | ^21^ |
| 22 | Hierarchical deep compartment modeling: A workflow to leverage machine learning and Bayesian inference for hierarchical pharmacometric modeling | Clinical Development/Precision Medicine | Introduces hierarchical deep compartment modeling (HDCM), combining machine learning (deep neural networks) and Bayesian inference for automated, flexible covariate modeling in hierarchical pharmacometric population PK analyses. | HDCM extends deep compartment modeling by modeling random effects and quantifying uncertainty; implemented in Julia, the workflow integrates a neural network for learning relationships between covariates and PK parameters, Bayesian inference for uncertainty, SHAP analysis for interpretability, and open-source code for reproducible population PK modeling and simulation using synthetic data. | Neural networks and SHAP analysis enable automated detection and interpretation of nonlinear covariate effects in PK modeling, while Bayesian ML approaches allow scalable uncertainty quantification, thus facilitating robust, explainable pharmacometric modeling in drug development and real-world studies | ^22^ |
| 23 | Pharmacogenomic augmented machine learning in electronic health record alerts: A health system-wide usability survey of clinicians | Clinical Development/Precision Medicine | Evaluates clinician usability and preferences for pharmacogenomic-augmented, machine learning-driven EHR drug alerts versus generic alerts, using a health systemwide survey to identify principles for designing actionable, less stressful digital alerts. | Clinicians strongly preferred simplified, patient-specific alerts using ML/AI-driven pharmacogenomic predictions over generic warnings; usability was consistently highest for concise, individualized efficacy alerts, with stress and alert burden decreased by actionable, relevant communication—preferences varying by specialty and clinical experience | Machine learning models integrating pharmacogenomic and clinical variables can generate EHR-embedded drug response predictions, supporting the development of adaptive, user-centered digital alerts that enhance clinical decision-making and reduce burnout in precision medicine workflows | ^23^ |
| 24 | Computational drug discovery pipelines identify NAMPT as a therapeutic target in neuroendocrine prostate cancer | Clinical Development/Precision Medicine | Designs and validates a computational drug discovery pipeline that identifies NAMPT as an actionable therapeutic target in neuroendocrine prostate cancer (NEPC), using multi-cohort transcriptomic profiling and in vitro validation. | NAMPT inhibitors showed robust efficacy in NEPC cell models, outperforming controls, and a causal biomarker discovery approach (PC-simple) was implemented to identify and validate genes directly linked to drug sensitivity, which aid patient selection and clinical trial design for NAMPT-based therapy | The workflow demonstrates use of causal inference, graph algorithms, and predictive modeling to automate drug candidate selection and biomarker identification, enabling rapid repurposing and precision targeting in complex, rare cancer populations | ^24^ |
| 25 | Explainable machine learning prediction of edema adverse events in patients treated with tepotinib | Clinical Development/Precision Medicine | Applies machine learning methods (random forest and gradient boosting) to predict the incidence and severity of edema adverse events in tepotinib-treated cancer patients and identifies critical contributing factors using explainability techniques | The best model (random forest with engineered longitudinal covariates) had high predictive accuracy (weighted F1 score up to 0.961); SHAP analysis revealed serum albumin and age as major drivers of risk, and dose modifications were linked to mitigating edema, providing interpretable insights for patient-level safety management | The presented framework enables systematic risk prediction and individualized safety monitoring for drug-induced edema using ML models, with explainable outputs that can support decision-making, trial enrichment, dose modification strategies, and future longitudinal safety modeling in oncology | ^25^ |
| 26 | From organs to algorithms: Redefining cancer classification in the age of artificial intelligence | Clinical Development/Precision Medicine | Advocates for a transformation in cancer classification—from organ/histology-based methods toward AI-driven, multimodal integration of molecular, imaging, histopathologic, and clinical data to achieve precision oncology. | AI tools—including deep learning and radiomics—can standardize diagnosis, accelerate workflow, and uncover novel features or subtypes overlooked by traditional classification; multimodal approaches using CNNs, autoencoders, and graph neural networks reveal new phenotypes, prognostic indicators, and biologically meaningful subgroupings directly linked to treatment outcomes | AI-powered workflows that combine digital pathology, quantitative imaging, multi-omics, and clinical records enable granular reclassification, biomarker discovery, and individualized treatment selection for cancer patients, moving closer to the vision of fully personalized, mechanism-based medicine | ^26^ |
| 27 | Increasing acceptance of AI-generated digital twins through clinical trial applications | Clinical Development/Precision Medicine | Discusses strategies to increase clinical and regulatory acceptance of AI-generated digital twins for patients in clinical trials, proposing that leveraging these twins within regulated trials will accelerate their future adoption in clinical practice | AI-generated digital twins can simulate individual patient trajectories using multimodal data, enabling virtual control groups, variance reduction, and more flexible trial designs (PROCOVA); the European Medicines Agency and FDA are already formalizing guidance for prognostic covariate adjustment, and explainability (via SHAP, LIME) and robust validation are emphasized as necessary for trust and adoption | Digital twins generated by AI can power in silico clinical trials, synthetic controls, prognostic risk scoring, and adaptive inclusion criteria, ultimately reducing trial cost and duration while facilitating the transition to personalized medicine through transparent and validated virtual patient modeling | ^27^ |
| 28 | Accelerating healthcare innovation: the role of Artificial intelligence and digital health technologies in critical path institute’s public‐private partnerships | Clinical Development/Precision Medicine | Reviews how public-private partnerships, specifically Critical Path Institute, drive AI and digital health technology (DHT) innovation in drug development through regulatory-endorsed collaboration, data harmonization, and scalable infrastructure. | C-Path and partners power FDA/EMA-endorsed initiatives using cloud-based data pipelines, machine learning, synthetic patient generation, wearable sensor analytics, and real-world data frameworks for disease monitoring and clinical trial enrichment, while maintaining patient privacy and process validation | Scalable, interpretable AI models and NLP streamline regulatory submissions, trial simulation, risk scoring, and digital biomarker development; public-private frameworks foster evidence-based validation and adoption of AI and DHT tools across the drug development lifecycle | ^28^ |
| 29 | Potential Meets Practicality: AI's Current Impact on the Evidence Generation and Synthesis Pipeline in Health Economics | Post-Marketing, Safety, and Real-World Implementation | Review and discuss how in silico and artificial intelligence models can advance the prediction of drug disposition and response across diverse populations and life stages in drug development | Summarizes applications of PBPK and QSP/QST modeling for virtual populations, presents a case study in drug-induced liver injury for postmenopausal women, and discusses strategies and challenges for integrating AI with modeling to improve drug development and dosing predictions. | AI methods, such as machine learning and generative adversarial networks, are highlighted for analyzing real-world data, creating virtual populations, and identifying physiological determinants to enhance drug exposure and outcome predictions, particularly for underrepresented patient groups | ^29^ |
| 30 | Rapid identification and phenotyping of nonalcoholic fatty liver disease patients using a machine-based approach in diverse healthcare systems | Post-Marketing, Safety, and Real-World Implementation | Presents a machine-based phenotyping algorithm utilizing EHR data to rapidly identify and stratify nonalcoholic fatty liver disease (NAFLD) patients in diverse healthcare systems, aiming to enable timely intervention and risk assessment beyond conventional diagnosis code approaches | The algorithm combines structured and unstructured EHR data (codes, labs, notes) and noninvasive fibrosis scoring, achieving high performance (mean PPV 85%, sensitivity 80%), discovering thousands of previously undiagnosed NAFLD cases across three major health centers, and outperforming simpler rule-based methods for risk stratification and phenotyping | Leveraging rule-based algorithms and natural language processing for scalable cohort discovery, automated clinical risk stratification, and future integration with ML and biomarker data to improve real-world NAFLD screening, trial selection, and personalized disease management in large EHR settings | ^30^ |
| 31 | Real-world evidence in the cloud: Tutorial on developing an end-to-end data and analytics pipeline using Amazon Web Services resources | Post-Marketing, Safety, and Real-World Implementation | Demonstrates how to build an end-to-end cloud-based data and analytics pipeline for real-world evidence (RWE) generation in drug development, exemplified by the CURE ID platform using Amazon Web Services resources | The pipeline includes data ingestion, transformation (including NLP), visualization, and analytics modules, leverages AWS Lambda, RDS, QuickSight, and SageMaker for scalable processing of multimodal real-world data, and enables advanced analyses like topic modeling and clustering for drug repurposing and treatment pattern discovery | The architecture supports integration of machine learning and natural language processing to automate cohort identification, risk assessment, and mining of unstructured clinical text, making advanced, scalable AI-driven analytics possible across diverse patient populations and use cases in biomedical research | ^31^ |

*Research conducted in several manuscripts spans multiple stages of drug development. The listed stage reflects the closest match aligned with the main research focus, based on the authors’ judgement.

**References**

1. Walter, M.*, et al*. Predicting Pharmacokinetics in Rats Using Machine Learning: A Comparative Study Between Empirical, Compartmental, and PBPK-Based Approaches. *Clin Transl Sci*. **18**, e70150 (2025).

2. Lu, J.*, et al*. Large Language Models and Their Applications in Drug Discovery and Development: A Primer. *Clin Transl Sci*. **18**, e70205 (2025).

3. Kanakia, A., Sale, M., Zhao, L. & Zhou, Z. AI In Action: Redefining Drug Discovery and Development. *Clin Transl Sci*. **18**, e70149 (2025).

4. Titar, R.R. & Ramanathan, M. Variational autoencoders for generative modeling of drug dosing determinants in renal, hepatic, metabolic, and cardiac disease states. *Clin Transl Sci*. **17**, e13872 (2024).

5. Pillai, N., Abos, A., Teutonico, D. & Mavroudis, P.D. Machine learning framework to predict pharmacokinetic profile of small molecule drugs based on chemical structure. *Clin Transl Sci*. **17**, e13824 (2024).

6. Hämäläinen, K.*, et al*. Non-targeted metabolomics for the identification of plasma metabolites associated with organic anion transporting polypeptide 1B1 function. *Clin Transl Sci*. **17**, e13773 (2024).

7. Zetterman, T., Nieminen, A.I., Markkula, R., Kalso, E. & Lötsch, J. Machine learning identifies fatigue as a key symptom of fibromyalgia reflected in tyrosine, purine, pyrimidine, and glutaminergic metabolism. *Clin Transl Sci*. **17**, e13740 (2024).

8. Yang, K., Gonzalez, D., Woodhead, J.L., Bhargava, P. & Ramanathan, M. Leveraging In Silico and Artificial Intelligence Models to Advance Drug Disposition and Response Predictions Across the Lifespan. *Clin Transl Sci*. **18**, e70272 (2025).

9. Shahin, M.H.*, et al*. AI-Driven Applications in Clinical Pharmacology and Translational Science: Insights From the ASCPT 2024 AI Preconference. *Clin Transl Sci*. **18**, e70203 (2025).

10. Lan, J.*, et al*. MoLPre: A Machine Learning Model to Predict Metastasis of cT1 Solid Lung Cancer. *Clin Transl Sci*. **18**, e70186 (2025).

11. Wiens, M., Verone-Boyle, A., Henscheid, N., Podichetty, J.T. & Burton, J. A Tutorial and Use Case Example of the eXtreme Gradient Boosting (XGBoost) Artificial Intelligence Algorithm for Drug Development Applications. *Clin Transl Sci*. **18**, e70172 (2025).

12. Huh, K.Y.*, et al*. Exploration of Using an Open-Source Large Language Model for Analyzing Trial Information: A Case Study of Clinical Trials With Decentralized Elements. *Clin Transl Sci*. **18**, e70183 (2025).

13. Shahin, M.H., Goswami, S., Lobentanzer, S. & Corrigan, B.W. Agents for Change: Artificial Intelligent Workflows for Quantitative Clinical Pharmacology and Translational Sciences. *Clin Transl Sci*. **18**, e70188 (2025).

14. Gomeni, R. & Bressolle-Gomeni, F. Comparison of Different Machine Learning Methodologies for Predicting the Non-Specific Treatment Response in Placebo Controlled Major Depressive Disorder Clinical Trials. *Clin Transl Sci*. **18**, e70128 (2025).

15. Raman, K., Kumar, R., Musante, C.J. & Madhavan, S. Integrating Model-Informed Drug Development With AI: A Synergistic Approach to Accelerating Pharmaceutical Innovation. *Clin Transl Sci*. **18**, e70124 (2025).

16. Weng, C., Lin, W., Dong, S., Liu, Q. & Zhang, H. First, Do No Harm: Addressing AI's Challenges With Out-of-Distribution Data in Medicine. *Clin Transl Sci*. **18**, e70132 (2025).

17. Zhang, J.*, et al*. Establishment and Validation of a Machine-Learning Prediction Nomogram Based on Lymphocyte Subtyping for Intra-Abdominal Candidiasis in Septic Patients. *Clin Transl Sci*. **18**, e70140 (2025).

18. Ambe, K.*, et al*. Prediction of Cisplatin-Induced Acute Kidney Injury Using an Interpretable Machine Learning Model and Electronic Medical Record Information. *Clin Transl Sci*. **18**, e70115 (2025).

19. Venkatapurapu, S.P., Gibbs, M. & Kimko, H. Augmented intelligence in precision medicine: Transforming clinical decision-making with AI/ML and/or quantitative systems pharmacology models. *Clin Transl Sci*. **17**, e70112 (2024).

20. Bernal, E.A., Yang, S., Herbst, K. & Venuto, C.S. Comparing machine learning and deep learning models to predict cognition progression in Parkinson's disease. *Clin Transl Sci*. **17**, e70066 (2024).

21. Ponce-Bobadilla, A.V., Schmitt, V., Maier, C.S., Mensing, S. & Stodtmann, S. Practical guide to SHAP analysis: Explaining supervised machine learning model predictions in drug development. *Clin Transl Sci*. **17**, e70056 (2024).

22. Elmokadem, A.*, et al*. Hierarchical deep compartment modeling: A workflow to leverage machine learning and Bayesian inference for hierarchical pharmacometric modeling. *Clin Transl Sci*. **17**, e70045 (2024).

23. Grant, C.W.*, et al*. Pharmacogenomic augmented machine learning in electronic health record alerts: A health system-wide usability survey of clinicians. *Clin Transl Sci*. **17**, e70044 (2024).

24. Zhang, W., Lee, A., Lee, L., Dehm, S.M. & Huang, R.S. Computational drug discovery pipelines identify NAMPT as a therapeutic target in neuroendocrine prostate cancer. *Clin Transl Sci*. **17**, e70030 (2024).

25. Amato, F.*, et al*. Explainable machine learning prediction of edema adverse events in patients treated with tepotinib. *Clin Transl Sci*. **17**, e70010 (2024).

26. Khozin, S. From organs to algorithms: Redefining cancer classification in the age of artificial intelligence. *Clin Transl Sci*. **17**, e70001 (2024).

27. Vidovszky, A.A.*, et al*. Increasing acceptance of AI-generated digital twins through clinical trial applications. *Clin Transl Sci*. **17**, e13897 (2024).

28. Podichetty, J.T.*, et al*. Accelerating healthcare innovation: the role of Artificial intelligence and digital health technologies in critical path institute's public-private partnerships. *Clin Transl Sci*. **17**, e13851 (2024).

29. Naylor, N.R., Hummel, N., de Moor, C. & Kadambi, A. Potential Meets Practicality: AI's Current Impact on the Evidence Generation and Synthesis Pipeline in Health Economics. *Clin Transl Sci*. **18**, e70206 (2025).

30. Basile, A.O.*, et al*. Rapid identification and phenotyping of nonalcoholic fatty liver disease patients using a machine-based approach in diverse healthcare systems. *Clin Transl Sci*. **18**, e70105 (2025).

31. Anderson, W.*, et al*. Real-world evidence in the cloud: Tutorial on developing an end-to-end data and analytics pipeline using Amazon Web Services resources. *Clin Transl Sci*. **17**, e70078 (2024).
